# Opportunities and challenges of phenomics applied to livestock and aquaculture breeding in South America

**DOI:** 10.1093/af/vfaa008

**Published:** 2020-04-01

**Authors:** Ricardo Vieira Ventura, Fabyano Fonseca e Silva, José Manuel Yáñez, Luiz F Brito

**Affiliations:** 1 Department of Animal Nutrition and Production, Faculty of Veterinary Medicine and Animal Science, University of São Paulo (FMVZ/USP), Pirassununga, SP, Brazil; 2 Department of Animal Science, Federal University of Viçosa, Viçosa, MG, Brazil; 3 Faculty of Veterinary and Animal Sciences, University of Chile, Santa Rosa, La Pintana, Santiago, Chile; 4 Department of Animal Sciences, Purdue University, West Lafayette, IN

**Keywords:** animal production, beef cattle, developing countries, salmon, tropical livestock

**Implications**

- South America is a major food producer in the world, but there is still a large potential to optimize production efficiency and the industries’ long-term sustainability through the incorporation of modern phenotyping technologies, advanced breeding schemes, and better management strategies.
- There is a great diversity of livestock production systems (e.g., pasture-based, mountainous regions, feedlots) in South America, which makes it more challenging to collect certain phenotypes and also requires accounting for genotype-by-environment interactions in genetic evaluation models.
- There is a lack of large-scale and well-structured breeding programs for the majority of livestock and aquaculture species, mainly due to the low investments in pedigree recording, genotyping, and high-throughput phenotyping in commercial and small holder farms.
- As phenotype and pedigree recording and genotyping are still challenging in South America, data sharing across breeding associations and research institutions is paramount, especially for building training populations for genomic selection.

## Introduction

The world population is expected to reach 9.1 billion people by 2050 (FAO, 2019) and the largest area of populational growth is projected to occur in developing countries. Consequently, there is an urgent need to increase food production in these regions based on an efficient exploitation of a wide range of genetic resources (species, breeds) and production systems. In this context, South America is one of the major livestock and aquaculture producers in the world, especially beef, pork, poultry, South American camelids, tilapia, and salmon. As in other developing regions, the great productivity is accompanied by a large diversity of production systems (e.g., intensive, pasture-based, mountainous regions, and small holders), climatic and geographical conditions, availability of natural resources (e.g., water and forage), and genetic resources (numerous species, breeds, and populations). In order to meet the growing food demand and be competitive on a global scale, there is a need to increase production efficiency and therefore, long-term sustainability. This can be achieved through selective breeding by identifying animals with greater genetic merit for the traits of interest. Sustainable genetic improvement can only be achieved through accurate, routine recording, and proper use of at least phenotypic and pedigree information.

The success of selective breeding is highly dependent on the quality of the phenotypes recorded and their degree of representation of the biological and physiological mechanisms underlying the breeding goals of interest. Development of equipment and efficient measurement’ protocols (e.g., automatic milking systems, visual computing, infrared spectroscopy, biosensors and external sensors, and satellite-based images), the availability of new “-omics” technologies (e.g., genomics, metabolomics, proteomics, transcriptomics), and new statistical and bioinformatic tools, have enabled the livestock and aquaculture industries to speed up the rates of genetic progress per unit of time.

High-throughput phenotyping is a reality in livestock and aquaculture production systems in developed countries (e.g., McParland et al., 2016; Saberioon et al., 2017). However, numerous attempts to establish representative large-scale phenotype recording systems applied to breeding programs in South American countries have failed for various reasons. This includes poorly structured data collection systems, temporal discontinuity of data recording, extensive or small holder farms (more difficult to collect phenotypes), and disinterest or lack of financial resources from the industry to invest in large-scale and continuous phenotyping projects. Furthermore, the existence of genotype-by-environment interactions (GxE) plays a major role in the successful use of genetic material developed in other countries, which then requires the implementation of regional breeding programs. The main objectives of this paper are: 1) to succinctly review the current phenotypic collection systems coupled to breeding programs in South America, with a greater emphasis on Brazilian beef cattle and Chilean aquaculture; and 2) to present the opportunities and challenges in the area of phenomics to advance genetic improvement in livestock and aquaculture in South American countries.

## Beef Cattle

South America is a major beef cattle producer, with approximately 25% of the worldwide cattle population (FAOSTAT, 2017). The cattle industry is based on a plethora of systems varying from small-holder farmers with low-productivity levels, basic management issues (e.g., nutritional, disease control) and minimal use of technology, to very large producers with intensive adoption of precision livestock technologies. These production systems include divergent genetic resources, with a predominance of Zebu cattle (*Bos taurus indicus*) in tropical regions (e.g., Brazil, Paraguay, Colombia, Venezuela, Peru, and Bolivia) and Taurine breeds or crosses in sub-tropical regions (e.g., South of Brazil, Uruguay, Argentina, and Chile). The three main beef cattle producers in South America are Brazil, Argentina, and Uruguay. Beef cattle production is pasture-based, with certain regions also finishing animals in feedlots. Nellore (Zebu cattle) is the main beef breed raised in South America.

The main current breeding goals, according to its relative importance on economic indexes, are: growth (particularly growth rate and cow maturity weight) and reproduction. New initiatives are evaluating the inclusion of feed efficiency and meat/carcass quality (ultrasound-based carcass measurements) as index components. In order to meet such goals, there are around 10 Nellore breeding programs currently established in Brazil, evaluating over 0.5 million cows per year (Carvalheiro et al., 2014). There are breeding program initiatives for other breeds as well (e.g., Angus, Montana Tropical Composite, Braford, and Hereford). The data collection system in most breeding programs is mainly performed by technicians that periodically visit herds spread out across the country (mainly to evaluate animals by visual scoring), in addition to data transfer uploaded into independent databases (owned by each breeding program).

### Undergoing research in the area of beef cattle phenomics

As previously mentioned, improved feed efficiency is a key breeding goal. However, the cost and difficulty to quantify individual feed intake, especially in pasture-based systems, have limited the inclusion of this trait in breeding programs. Over the past decade, various research groups in South America have concentrated efforts on identifying the best indicator variables of feed efficiency, unveiling the genetic relationship with traits already evaluated, and investigating the feasibility of developing a training population for genomic selection (Santana et al., 2014; Silva et al., 2016). Recent studies have shown that a 35-d test with automatic tools for weight collection can attain sufficient phenotyping precision for traits considered in the Brazilian genetic evaluation of Nellore cattle (Torres-Junior et al., 2018).

Automated feeding systems have been implemented mainly in feedlots or research farms (www.intergado.com.br/intergado-efficiency/; https://growsafe.com/about/). For instance, data from experimental farms have been used to derive feed efficiency-related traits, such as RFI, feed conversion ratio, average daily gain, and dry-matter intake (Silva et al., 2016). Furthermore, these systems are usually coupled with other innovative tools (e.g., sensors and scan cameras) to measure (or predict) additional variables to be potentially included in breeding programs. These traits include in vivo carcass yield, body condition score, meat quality (Gomes et al., 2016), and behavioral traits (e.g., feeding behavior, temperament, and social dominance).

Over the past years, various studies have focused on identifying more specialized phenotypes related to reproductive traits. For instance, the number of antral follicles influences heifer pregnancy rate and the success of reproductive technologies (ovum pick-up, in vitro fertilization), which are widely used in Brazil (Oliveira-Junior et al., 2017). Testicular hypoplasia, a disorder attributed to incomplete development of the germinal epithelium of the seminiferous tubules has also been studied, which enabled the identification of important genomic regions affecting this reproductive disorder (Neves et al., 2019).

Animal resilience and adaptation is paramount in South American livestock production systems. For instance, tick infestation causes large economic losses and welfare issues, especially in Taurine populations. Therefore, infrared thermo-imaging was investigated as a potential tool to quantify (count) the number of ticks in the body surface of Brangus cattle (Barbedo et al., 2017). However, the results were not satisfactory as the suggested approach only captured a small contrast between ticks and the animal hair coat. Alternative adaptation traits include scoring (subjective) protocols for traits such as prepuce (navel) length, hair length, ocular pigmentation, and tick resistance (based on tick count in one side of the animal body). These indicator traits were shown to be heritable and can be implemented in genomic selection schemes (Piccoli et al., 2019).

Zebu breeds are known for having lower meat quality (marbling and tenderness) compared to Taurine animals. However, there is enough genetic variability to enable selective breeding (Magalhaes et al., 2018). As meat quality traits are usually difficult to measure (slaughter of potential selection candidates), genomic selection will play an important role. Furthermore, near-infrared reflectance spectroscopy (NIRS) has been investigated as a non-destructive alternative to predict meat quality traits in Nellore cattle (Magalhaes et al., 2018). NIRS was shown to be useful to predict tenderness and meat color, but not indicated for subjective traits such as marbling. The genomic background of meat fatty acid profiling has also been investigated as an additional breeding goal in Nellore (Lemos et al., 2016; Feitosa et al., 2019).

**Figure 1.**
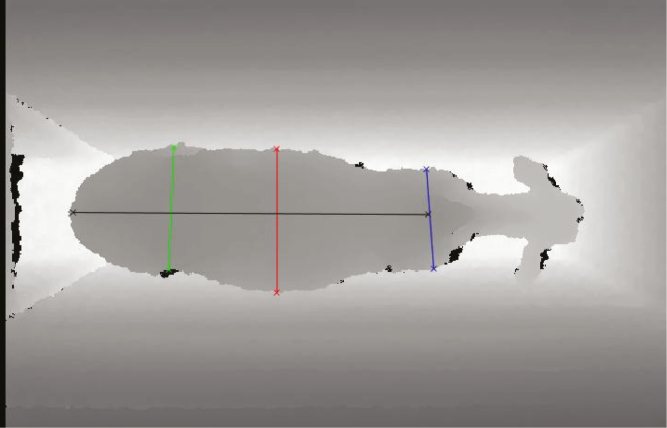
An example of body measurements taken from a Nellore calf using the Microsoft Kinect tool.

Better assessments of carcass yield and meat quality traits are still needed. Another potential alternative could be through collaborations with commercial slaughterhouses. However, there is limited interest from the beef industry on sharing and using such data for breeding purposes. In this context, image analysis and visual computing could be promising tools to predict carcass yield in different ages. Sensors (e.g., Microsoft Kinect—MK) can be applied for this purpose. MK can minimize imaging interferences due to the ambient light through deep mapping image technology (Gomes et al., 2016). Subsequently, as shown in Figure 1, these image sections from the width of chest, thorax, and abdomen; body length; and, dorsal height can be used to predict carcass yield in cattle (as in Nellore, Gomes et al., 2016).

A common challenge associated with pasture-based production systems is the long period of food scarcity caused by the seasonal patterns of rainfall, and high temperature and humidity. In this context, genetic selection for improved animal resilience and adaptation in such environments is gaining importance (Carvalheiro et al., 2019). Another major issue is GxE, especially because of the outdoor production systems (pasture- or feedlot-based). Additionally, the feasibility of using datasets recorded in a particular environment to improve the genomic predictive performance and subsequent selection of breeding bulls in untested environments has become an important topic of research (Cappa et al., 2017). For instance, one could be interested in using proven bulls from Argentina to breed (through artificial insemination—AI) with cows raised in harsh environmental conditions in Brazil. Environmental covariables can be used to predict the performance of individuals in untested locations. This can be achieved through the use of geographical information systems (GIS) as environmental index in GxE models. In our conceptualization of GxE based on environmental index obtained from GIS, a particular geographical area is considered as a geoprocessing environment corresponding to a grid of pixels, and for any single environmental variable, a value can be assigned to each pixel. The value distribution of a particular environmental variable in this set of pixels is termed “envirotype” for each geographical area (van Eeuwijk et al., 2019).

### Challenges and opportunities in beef cattle phenomics

There are several challenges for wider adoption of phenotyping tools in beef cattle, but at the same time, modern technologies are becoming more accessible and producers more interested in their adoption. For instance, Baruselli et al. (2017a) suggested a more aggressive strategy including the concentration of pregnancies early in the breeding season to improve reproductive efficiency. These authors and other research groups (e.g. Pugliesi et al., 2019) recommended the use of AI in all females at the beginning of the breeding season, and an early identification of non-pregnant cows, using color Doppler ultrasonography with subsequent AI.

**Figure 2.**
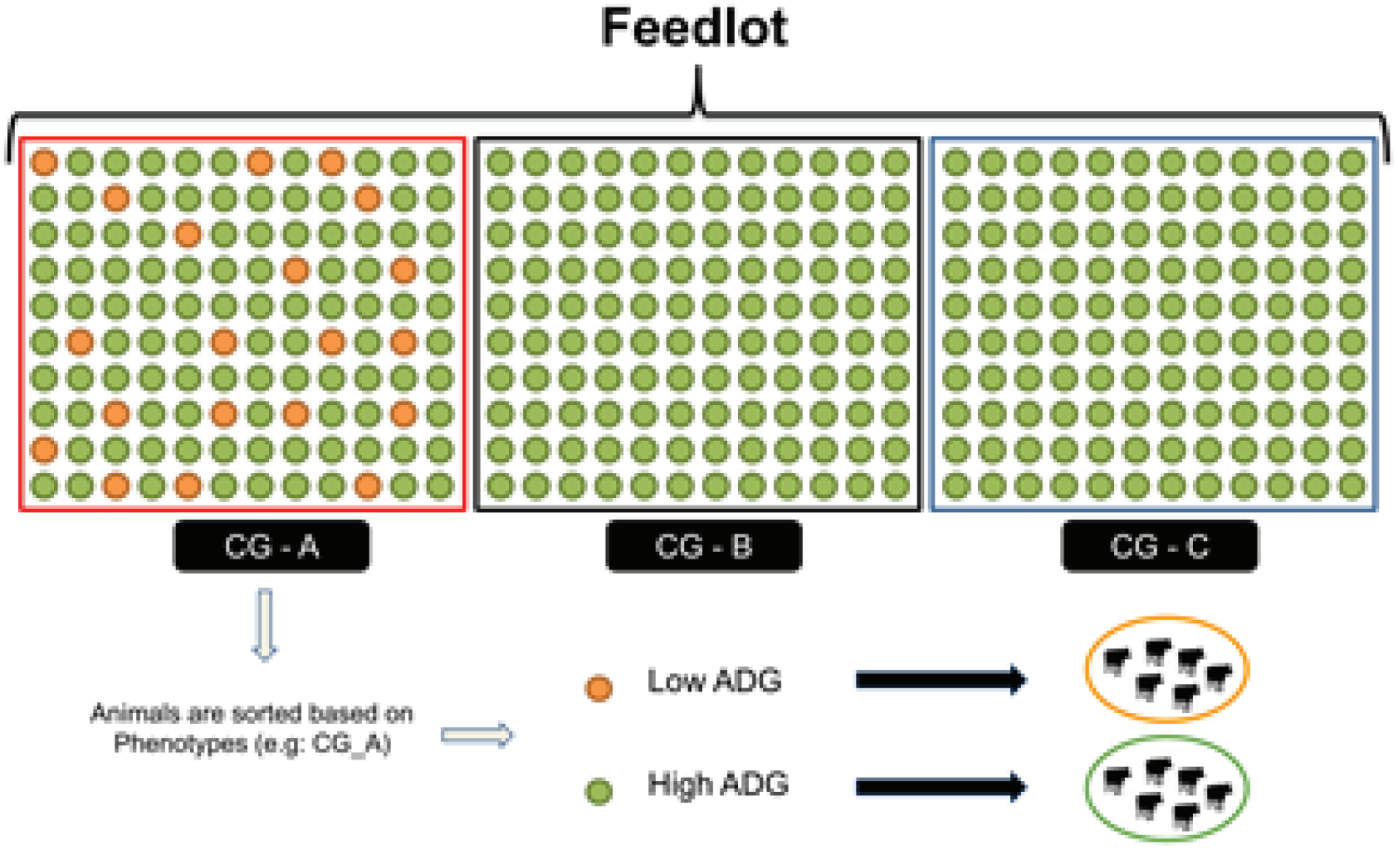
A scheme illustrating the phenotypic clustering of animals based on average daily gain. Biological samples from each group would be pooled together and genotyped as a single sample.

The DNA pooling technique (Bell et al., 2017) is another technology that could be used to build a training population for genomic selection and to identify genomic regions affecting fertility traits in beef cattle. This strategy requires the identification of animals with divergent phenotypes (e.g. females pregnant at the first compared to those nonpregnant after three rounds of AI) and the genotyping of pooled biological samples of females clustered in the same phenotypic group, instead of genotyping each single female. The DNA pooling strategy could be also applied for other purposes, such as in feedlot systems (phenotype extremes defined based on high or low growth rate; Figure 2). Furthermore, Brazilian start-up companies have recently been granted funding for the development of 3D cameras to predict cattle live weight, and to install automated scales and image analysis in feedlot operations to determine the optimal slaughter time (https://olhododono.agr.br/; https://techagr.com/). This could be used to identify the selection candidates (e.g., sires) more related to each DNA pool, assuming that all selection candidates are individually genotyped. Through this approach, phenotypes from commercial farms (or slaughter plants) could contribute to improve the performance of genomic evaluation for certain traits, as shown in Figure 2.

As there are limited financial resources and infrastructure for collecting phenotypes and individual genotyping in developing countries, the establishment of collaborations for data sharing is of utmost value. For instance, recent efforts in Brazil have been made to join existing genomic datasets for imputation purposes (https://bv.fapesp.br/pt/auxilios/97280). This will substantially reduce genotyping costs.

## Dairy Species

**Figure 3.**
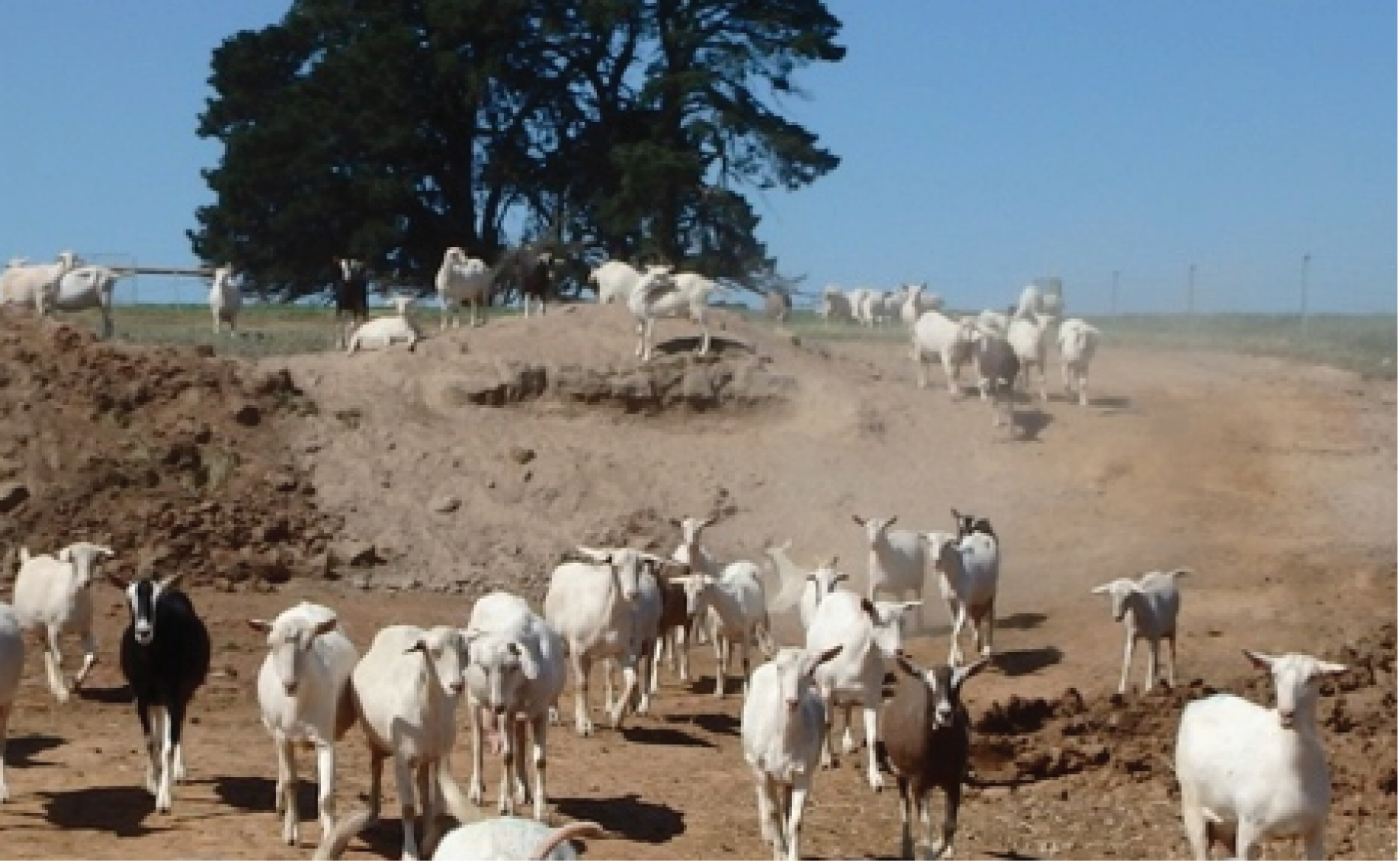
A dairy goats herd composed by Alpine and Saanen animals, which are raised in a semi-intensive production system.

Dairy cattle are the main milk producing species in South America, but sheep, goats, and buffaloes are also raised for similar purposes. Milk production in South American countries are mainly characterized by pasture-based production systems. However, an intensification trend has been observed after the inclusion of several large dairy operations, characterized by advances in nutrition and management practices. Girolando, a composite dairy breed developed in Brazil by crossing Holstein and Gyr cattle, is the main genetic resource used for milk production in the tropical regions of Brazil (Canaza-Cayo et al., 2018). Dairy goats (especially Alpine and Saanen breeds; Figure 3) have, to a lesser extent, economic importance in certain regions of South America, including the Northeast and Southeast of Brazil. Buffaloes are also raised for milk production (and cheese-making), especially in the Southeast and North of Brazil, Bolivia and Colombia. There are some dairy sheep producers, mainly in Argentina, Uruguay, Chile, and Brazil. However, these are usually small-holder farmers with no routine recording of phenotypes, with exception of a few research flocks.

Over the past decades, selection for improved milk production (total yield) was the main breeding goal, as the large majority of producers were paid only based on milk volume. Genetic improvement for additional traits was also achieved by importing semen from bulls (or other dairy species males) selected for additional traits including milk composition, conformation, health, and reproductive ability. In the past two decades, the breeding goals in South America have been refined to include other traits. Currently, the main trait categories are as follows: milk production and composition (especially fat and protein), udder health (based on somatic cell count), fertility and reproduction (e.g. age at first parity and interval between parities), workability (especially milking temperament in dairy cattle populations with genetic contribution from Zebu breeds), and conformation traits. For instance, the Brazilian Girolando and Gyr national breeding programs currently report breeding values for milking ease and temperament, 305-d milk yield and composition (fat, protein, and total solids), age at first calving and conformation. Adaptation and environmental resilience are also key breeding goals in dairy species raised in the tropical regions. The main indicator traits are heat tolerance, survival, and resistance to endo- and ectoparasites. However, genetic selection for these traits is still incipient.

Even though the breeding goals for dairy goats are similar to those described above, phenotypic selection for morphological traits was heavily weighted as there were no production or reproduction records and pedigree information. In Brazil, milk recording in dairy goats started only in 2005 (Facó et al., 2011). In addition to milk yield, other breeding goals include lactation length and reproductive traits (e.g., kidding interval and age at first kidding). The high adaptability of buffaloes to tropical regions has contributed to the population expansion in South America, especially in the Amazon region and Southern Brazil (>3 million animals). There are also some incipient breeding programs for buffaloes and the main breeding focus is milk yield, lactation persistency and milk composition.

### Research challenges and opportunities in dairy cattle phenomics

The dairy industry payment system is an important challenge for dairy improvement. For instance, mastitis (clinical and subclinical) is a great welfare and economic issue in South America (Goncalves et al., 2018) as producers are usually paid only based on total milk yield and penalized based on microbiological aspects of the milk. This has started to change in some regions, but still needs more research and investment. The usefulness of mid-infrared (MIR) analysis has been investigated as a phenotyping tool. For instance, Petrini et al. (2016) predicted milk components in Brazilian Holstein based on MIR and validated the results through gas chromatography. Matrix-assisted laser desorption/ionization time-of-flight mass spectrometry (MALDI-TOF) has been offered as a service by some laboratories and also at the research level as an alternative method for bacteria identification. On-farm culture systems, aiming to fast identify milk pathogens, have been implemented on several herds across South America.

**Figure 4.**
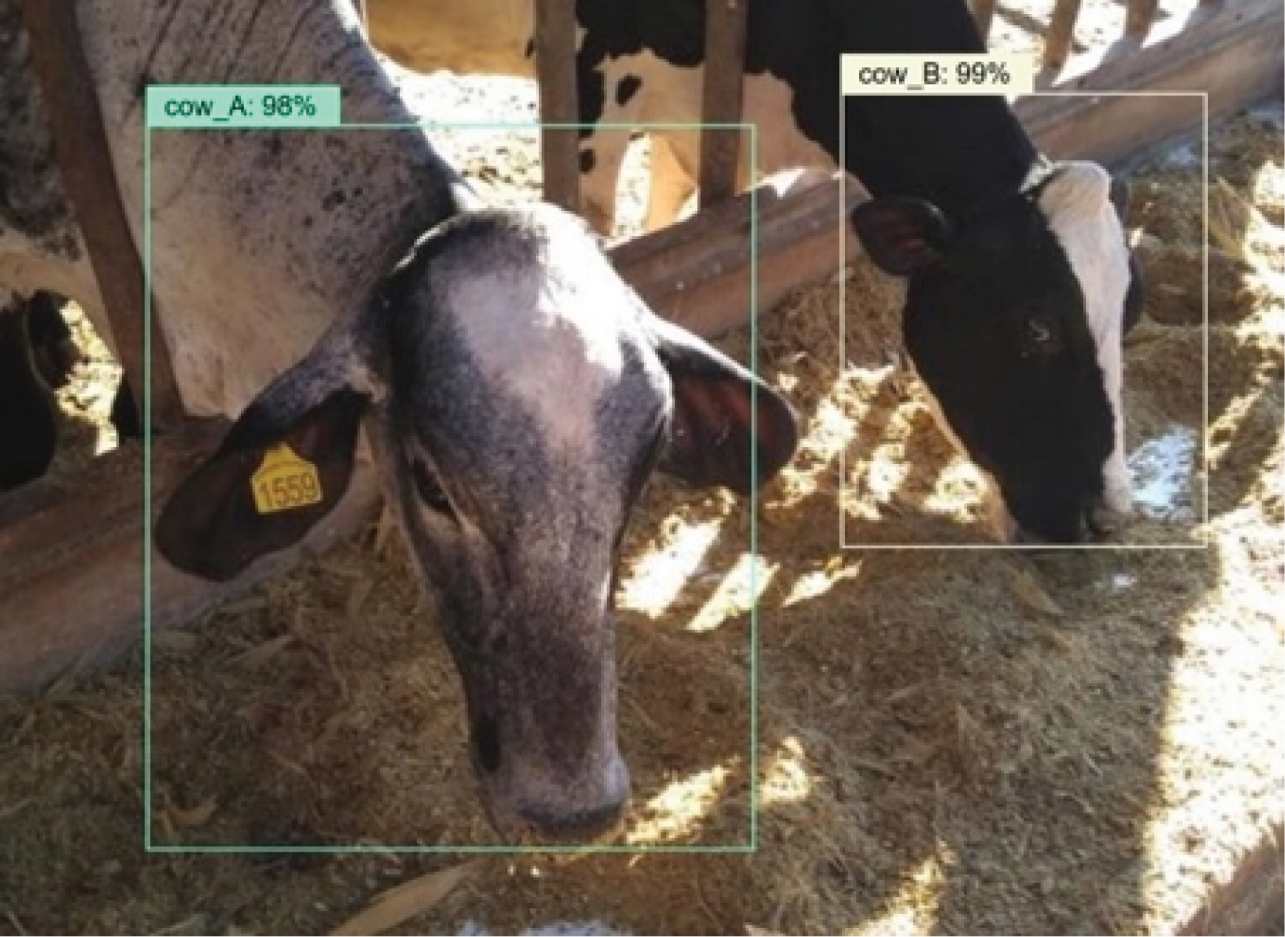
An example of computer vision use (pilot project) in which two cows were video identified with high similarity after an automated search in the image database. After identifying the animals, the data collected can be linked to their individual IDs.

Systems for monitoring individual feeding and drinking behavior have been recently tested in dairy cattle (e.g., Oliveira et al., 2018). The frequency and duration of each visit can be captured (feed and water), as well as feed and water intake. Correlations between the true data (obtained via video observation and manual weights) and predicted parameters were extremely high (0.917 for duration of feed visit, and >0.963 for all others). Computer vision is an expanding area in precision livestock, which enables identification of individual animals and posterior assigning of phenotypic records (e.g., eating time) to each individual (Figure 4).

There are several challenges involving dairy production in South America. First, there is a lack of centralized institutions integrating on-farm data collection, laboratory analysis of milk samples, and technical assistance to producers. Second, similarly to other regions around the world, the low profit margin and unstable milk prices make it difficult for producers to commit to investments in data recording. In most large dairy operations, protein and fat are usually controlled at the bulk tank level and not individually per cow. Somatic cell score, as part of a management protocol, is generally evaluated once a month in most herds. The lack of adequate payment and incentive to producers with regards to milk composition does not incentivize phenotyping and selection for such traits. However, there are some cases of success regarding data collection and genetic evaluations. For instance, the Holstein Association of Paraná (Parana, Brazil; www.apcbrh.com.br) provides a well-organized infrastructure to coordinate data collection, including laboratory milk analysis and genetic evaluations.

## Aquaculture

The aquaculture species with more advancements in terms of the implementation of genetic improvement approaches are salmonid and tilapia. The main trait included in the breeding goal for all aquaculture species is growth rate (Ponzoni et al., 2011; Lhorente et al., 2019). In addition, some other desirable traits are related with increased survival (resistance against viral, bacterial, and parasitic agents) and carcass-quality traits (fillet yield, fat content, and composition and flesh color; Gjedrem, 2012).

In general, growth-related traits can be directly measured in the selection candidates. However, disease resistance and carcass-quality traits are typically measured using sib-testing strategies (Yáñez et al., 2014). For instance, phenotypes for resistance to specific pathogens are usually recorded in highly controlled conditions using large-scale experimental challenges performed on thousands of full- and half-sibs of the selection candidates. Additionally, carcass-quality phenotypes are recorded in harvested fish at the processing plant using similar family-based designs (i.e., full- and half-sib testing).

The main drawback of phenotyping for disease resistance using experimental challenges is related to the correspondence between the traits measured in controlled versus field conditions, which might not be high enough, thus decreasing the rate of genetic progress achievable. In addition, the high cost associated with these experiments needs to be considered. With regards to the traits measured in processing plants, these are typically obtained using nonautomated measurements, which makes the procedures very labor-intensive, and in some cases biased due to human error. Therefore, the main opportunities for high-throughput phenotyping in aquaculture are related to the automation of phenotyping procedures. For instance, technologies combining automated image recording and computational algorithms that can predict individual body mass, sanitary condition (e.g., quantifying skin lessons and ectoparasite counting) and survival status from field conditions (i.e., sea sites, ponds, and tanks) will allow recording of tens of thousands of phenotypes per day (Føre et al., 2018). This will not only allow capture of longitudinal phenotypic data for growth and disease resistance traits but also recording data in the same environment in which the fishes are actually produced. Similar image-based approaches can be used at the processing plant to avoid manual manipulation and recording, aiming at having highly automated and digital phenotypes for carcass quality traits. Altogether, these approaches will improve accuracy and repeatability of phenotypic measurements, allowing continuous monitoring in the field and larger sample sizes, with a positive impact on fish welfare given a reduced need for fish management and experimental trials.

## Other Species

The majority of swine and poultry raised in commercial farms in South America are from genetic resources developed in other countries, especially in North America and Europe. For minor species (e.g., quail in Brazil), there are examples of ongoing breeding program initiatives and national technologies mainly related with automatic feeding intake (to quantify feed efficiency). According to Caetano et al. (2017a), improving feed conversion by identifying animals that require the same amount of feed but have higher body weight gain is key in modern quail breeding. Feeding represents the main production cost in meat quail (Caetano et al., 2017b), especially as protein is the highest cost component in their diets. Therefore, one possible strategy is to select quails based on their genetic performance over different protein levels in the diet. In summary, the investments in producing automatic feeders for quail is a good example of measuring novel traits with high economic value.

Many international genetic companies have applied modern phenotyping tools to obtain information on heat stress and behavior in pigs. This information is used at nucleus herds to obtain complementary traits when calculating selection indexes. The success of this strategy is maximized by using genomic information from both nucleus and commercial animals. In addition to the species mentioned above, meat sheep and goats, and South American camelids (mainly alpacas and llamas) are also of considerable importance to livestock production in South America. This is especially found in Uruguay, Southern Brazil, and Argentina (largest meat sheep producer region in South America), small-holder farmers in the Northeast and Central regions of Brazil (meat sheep and goats), and in the Andes Mountain region in Bolivia and Peru (small camelids). The main breeding goals in meat or dual-purpose (meat and wool) sheep include the following: wool (or fiber) weight and quality (e.g., dirty fleece weight, fiber diameter, clean fleece weight, and length of wick), body weight and growth rate (birth weight, weaning weight, and mature weight), carcass (e.g., loin-eye area, backfat thickness, leg score, primal cuts, and carcass weight), reproductive (e.g., litter size, age at first lambing, and lambing interval), and resilience (e.g., endoparasite resistance, disease resistance, maternal behavior, survival, longevity) traits. Meat sheep production systems in South America usually have low adoption of technologies with reduced to no phenotype recording, with the exception of Uruguay, Argentina, and some isolated regions of Brazil. In Brazil, there is a large number of locally developed sheep (e.g., Santa Ines hair sheep) and meat goats (e.g. Moxoto) breeds. The meat goat breeding goals are similar to those described above for meat sheep, with exception of wool/fiber traits. Phenotypic and pedigree recording in small ruminants is still limited in South America, but there are some breeding program initiatives with recent success.

## Conclusions

South America is a major livestock and aquaculture producer in the world. However, production efficiency is still lower than what could be achieved. This is partially due to the reduced adoption of phenotyping technologies, advanced breeding schemes and efficient management strategies. Furthermore, there is a great diversity of production systems, which makes it more challenging to collect certain phenotypes and also requires accounting for GxE interactions in genetic evaluation models. More recently, governments and industries are investing more in research and extension activities and there are clearer breeding goals, but the organization of data collection schemes and data sharing still needs to be substantially improved. We expect that phenomics will play an important role in improving livestock and aquaculture production efficiency in South America over the next few decades, considering that the industry continues (or increases) investments in phenotyping, pedigree recording and genotyping.

## About the Authors

**Ricardo Vieira Ventura** is a Professor in Bioinformatics Applied to Veterinary Medicine in the Department of Animal Nutrition and Production at the University of Sao Paulo (USP, Pirassununga, SP, Brazil). Dr. Ventura’s main research interests are software development applied to genetics and genomics, genomic selection, microbiome, computer vision and machine learning. **Corresponding author:** rvventura@usp.br

****

**Fabyano Fonseca e Silva** has a PhD in Statistical Sciences and is an Associate Professor in the Department of Animal Science at Universidade Federal de Viçosa (Brazil). His current research involves the use of Statistical Genomics and Bioinformatics in Animal Breeding, mainly applied to high-throughput phenotypes.

****

**José Manuel Yáñez** is an Associate Professor and Director of Research and Development at the Faculty of Veterinary and Animal Sciences, University of Chile (Santiago, Chile). Dr Yáñez is leading a research group devoted to aquaculture genetics and genomics, which is mainly focused on exploiting genomic information to implement and enhance new strategies to sustainably improve aquaculture production by means of selective breeding. He has a strong link to the fish farming industry and extensive experience as scientific and technical advisor in aquaculture breeding of different species including salmon, trout, tilapia, and other emerging species.

****

**Luiz F. Brito** is an Assistant Professor of Quantitative Genetics and Genomics in the Department of Animal Sciences at Purdue University (Indiana). He also holds an Adjunct Faculty position in the Department of Animal Biosciences at the University of Guelph (Canada). Luiz earned a BSc degree (with distinction) in Animal Sciences and a MSc in Genetics and Animal Breeding at the Federal University of Viçosa (Brazil). He completed his PhD and a postdoctoral fellowship at the Centre for Genetic Improvement of Livestock, University of Guelph (Canada). Luiz is broadly interested in quantitative genetics, genomics, and systems biology applied to genetic improvement of livestock species. His research group focuses on two main areas: 1) genetics and genomics of complex traits associated with animal behavior and welfare, environmental efficiency, and adaptation to challenging environments; and 2) development of genomic approaches to enable genetic improvement of minor species and small-holder livestock populations.

## References

[CIT0001] Barbedo J.G.A., Gomes C.C.G., Cardoso F.F., Domingues R., Ramos J.V., and McManus C.M. 2017 The use of infrared images to detect ticks in cattle and proposal of an algorithm for quantifying the infestation. Vet. Parasit. 235:106–112. doi:10.1016/j.vetpar.2017.01.020. 28215860

[CIT0002] Baruselli P. S., Ferreira R.M., Colli M.H.A., Elliff F.M., Sá Filho M.F., Vieira L., and Freitas B.G. 2017a Timed artificial insemination: current challenges and recent advances in reproductive efficiency in beef and dairy herds in Brazil. Anim. Reprod. 14:558–571. doi:10.21451/1984-3143-AR999.

[CIT0003] Bell A.M., Henshall J.M., Porto-Neto L.R., Dominik S., McCulloch R., Kijas J., and Lehnert S.A. 2017 Estimating the genetic merit of sires by using pooled DNA from progeny of undetermined pedigree. Genet. Sel. Evol. 49:28. doi:10.1186/s12711-017-0303-8. 28245804 PMC5331749

[CIT0004] Caetano G. C., Silva F.F., Calderano A.A., Silva L.P., Ribeiro J.C., Oliveira L.T.T., and Mota R.R. 2017 Genotype and protein level interaction in growth traits of meat-type quail through reaction norm models. J. Animal Feed Sci. 26:333–338. doi:10.22358/jafs/79806/2017.

[CIT0005] Caetano G. C., Silva D.A., Mota R.R., Oliveira H.R., Siqueira O.H.G.B.D., Resende M.D.V., Hannas M.I., and Silva F.F. 2017 Bayesian estimation of genetic parameters for individual feed conversion and body weight gain in meat quail. Livest. Sci. 200:76–79. doi:10.1016/j.livsci.2017.04.011.

[CIT0006] Canaza-Cayo A. W., Lopes P.S., Cobuci J.A., Martins M.F., and Silva M.V.G.B.D. 2018 Genetic parameters of milk production and reproduction traits of Girolando cattle in Brazil. Italian J. Animal Sci. 17(1):22–30. doi:10.1080/1828051X.2017.1335180.

[CIT0007] Cappa E. P., El-Kassaby Y.A., and Munoz F. 2017 Improving accuracy of breeding values by incorporating genomic information in spatial-competition mixed models. Mol. Breed. 37:125. doi:10.1007/s11032-017-0725-6.

[CIT0008] Carvalheiro R 2014 Genomic selection in Nelore cattle in Brazil. In: Proceedings of the 10th World Congress on Genetics Applied to Livestock Production; Vancouver, Canada; p. 17–22.

[CIT0009] Carvalheiro R., Costilla R., Neves H.H.R., Albuquerque L.G., Moore S., and Hayes B.J. 2019 Unraveling genetic sensitivity of beef cattle to environmental variation under tropical conditions. Genet. Sel. Evol. 51:29. doi:10.1186/s12711-019-0470-x. 31221081 PMC6585094

[CIT0010] van Eeuwijk F.A., Bustos-Korts D., Millet E.J., Boer M.P., Kruijer W., Thompson A., Malosetti M., Iwata H., Quiroz R., Kuppe C., et al. 2019 Modelling strategies for assessing and increasing the effectiveness of new phenotyping techniques in plant breeding. Plant Sci. 282:23–39. doi:10.1016/j.plantsci.2018.06.018. 31003609

[CIT0037] FAO. 2019 How to feed the world in 2050. [accessed November 25, 2019]. http://www.fao.org/fileadmin/templates/wsfs/docs/expert_paper/How_to_Feed_the_World_in_2050.pdf.

[CIT0038] FAOSTAT. 2017 Live animals. http://www.fao.org/faostat/en/#data/QA.

[CIT0011] Facó O., Lôbo R.N.B., Gouveia A.G., Guimaraes M.P., Fonseca J.F., Santos T.N.M., Silva M.A.A., and Villela L.C.V. 2011 Breeding plan for commercial dairy goat production systems in southern Brazil. Small. Rumin. Res. 98:164–169. doi:10.1016/j.smallrumres.2011.03.034.

[CIT0036] Feitosa F.L.B., Pereira A.S.C., Amorim S.T., Peripolli E., Silva R.M.D.O., Braz C.U., Ferrinho A.M., Schenkel F.S., Brito L.F., Espigolan R., et al 2019 Comparison between haplotype‐based and individual snp‐based genomic predictions for beef fatty acid profile in Nelore cattle. J. Anim. Breed Genet. 1–9. 10.1111/jbg.12463. 31867831

[CIT0012] Føre M., Frank K., Norton T., Svendsen E., Alfredsen J.A., Dempster T., Berckmans D. 2018 Precision fish farming: a new framework to improve production in aquaculture. Biosyst. Eng. 173:176–193. doi:10.1016/j.biosystemseng.2017.10.014.

[CIT0013] Gjedrem T 2012 Genetic improvement for the development of efficient global aquaculture: a personal opinion review. Aquaculture 344–349:12–22. doi:10.1016/j.aquaculture.2012.03.003.

[CIT0014] Gomes R.A., Monteiro G.R., Assis G.J., Busato K.C., Ladeira M.M., and Chizzotti M.L. 2016 Technical note: estimating body weight and body composition of beef cattle trough digital image analysis. J. Anim. Sci. 94:5414–5422. doi:10.2527/jas.2016-0797. 28046161

[CIT0015] Gonçalves J. L., Kamphuis C., Martins C.M., Barreiro J.R., Tomazi T., Gameiro A.H., Hogeveen H., and Santos M.V. 2018 Bovine subclinical mastitis reduces milk yield and economic return. Livest. Sci. 210:25–32. doi:10.1016/j.livsci.2018.01.016.

[CIT0016] Lemos M. V., Chiaia H. L., Berton M. P., Feitosa F. L., Aboujaoud C., Camargo G. M., Pereira A. S., Albuquerque L. G., Ferrinho A. M., Mueller L. F., et al. 2016 Genome-wide association between single nucleotide polymorphisms with beef fatty acid profile in Nellore cattle using the single step procedure. BMC Genomics 17:213. doi:10.1186/s12864-016-2511-y. 26960694 PMC4784275

[CIT0017] Lhorente J.P., Araneda M., Neira R., and Yáñez J.M. 2019 Advances in genetic improvement for salmon and trout aquaculture: the Chilean situation and prospects. Rev. Aquac. 11(2):340–353. doi:10.1111/raq.12335.

[CIT0018] Maciel I. C. F., Barbosa F. A., Tomich T. R., Ribeiro L. G. P., Alvarenga R. C., Lopes L. S., Malacco V. M. R., Rowntree J. E., Thompson L. R., and Lana Â. M. Q. 2019 Could the breed composition improve performance and change the enteric methane emissions from beef cattle in a tropical intensive production system? PLoS One 14:e0220247. doi:10.1371/journal.pone.0220247. 31348816 PMC6660127

[CIT0019] Magalhães A.F.B., Teixeira G.H.A., Ríos A.C.H., Silva D.B.D.S., Mota L.F.M., Muniz M.M.M., de Morais C.L.M., de Lima K.M.G., Cunha Júnior L.C., Baldi F., et al. 2018 Prediction of meat quality traits in Nelore cattle by near-infrared reflectance spectroscopy. J. Anim. Sci. 96:4229–4237. doi:10.1093/jas/sky284. 30010881 PMC6162584

[CIT0020] McParland S., and Berry D. P. 2016 The potential of Fourier transform infrared spectroscopy of milk samples to predict energy intake and efficiency in dairy cows. J. Dairy Sci. 99:4056–4070. doi:10.3168/jds.2015-10051. 26947296

[CIT0021] Neves H. H. R., Vargas G., Brito L. F., Schenkel F. S., Albuquerque L. G., and Carvalheiro R. 2019 Genetic and genomic analyses of testicular hypoplasia in Nellore cattle. PLoS One 14:e0211159. doi:10.1371/journal.pone.0211159. 30677076 PMC6345487

[CIT0022] Oliveira B. R., Ribas M. N., Machado F. S., Lima J. A. M., Cavalcanti L. F. L., Chizzotti M. L., and Coelho S. G. 2018 Validation of a system for monitoring individual feeding and drinking behaviour and intake in young cattle. Animal 12:634–639. doi:10.1017/S1751731117002002. 28820079

[CIT0023] Oliveira-Júnior G. O., Perez B.C., Cole J.B., Santana M.H.D. A., Silveira J., Mazzoni G., Ventura R.V., Júnior M.S., Kadarmideen H.N., Garrick D.J., et al 2017 Genomic study and Medical Subject Headings enrichment analysis of early pregnancy rate and antral follicle numbers in Nelore heifers. J. Animal Sci. 95(11):4796–4812. doi:10.2527/jas2017.1752. PMC6292327 29293733

[CIT0024] Petrini J., Iung L.H., Rodriguez M.A., Salvian M., Pértille F., Rovadoscki G.A., Cassoli L.D., Coutinho L.L., Machado P.F., Wiggans G.R., et al. 2016 Genetic parameters for milk fatty acids, milk yield and quality traits of a Holstein cattle population reared under tropical conditions. J. Anim. Breed. Genet. 133:384–395. doi:10.1111/jbg.12205. 26968150

[CIT0025] Piccoli M.L., Brito L.F., Braccini J., Oliveira H.R., Cardoso F.F., Roso V.M., Sargolzaei M., and Schenkel F.S. 2019 Comparison of genomic prediction methods for evaluation of adaptation and productive efficiency traits in Braford and Hereford cattle. Livest. Sci. 12:103864. doi:10.1016/j.livsci.2019.103864.

[CIT0026] Ponzoni R. W., Nguyen N.H., Khaw H.L., Hamzah A., Bakar K.R.A., and Yee H.Y. 2011 Genetic improvement of Nile tilapia (*Oreochromis niloticus*) with special reference to the work conducted by the World Fish Center with the GIFT strain. Rev. Aquac. 3(1):27–41. doi:10.1111/j.1753-5131.2010.01041.x.

[CIT0027] Pugliesi G., Bisinotto D. Z., Mello B. P., Lahr F. C., Ferreira C. A., Melo G. D., Bastos M. R., and Madureira E. H. 2019 A novel strategy for resynchronization of ovulation in Nelore cows using injectable progesterone (P4) and P4 releasing devices to perform two timed inseminations within 22 days. Reprod. Domest. Anim. 54:1149–1154. doi:10.1111/rda.13475. 31134689

[CIT0028] Saberioon M., Gholizadeh A., Cisar P., Pautsina A., and Urban J. 2017 Application of machine vision systems in aquaculture with emphasis on fish: state‐of‐the‐art and key issues. Rev. Aquac. 9:369–387. doi:10.1111/raq.12143.

[CIT0035] Santana M.H., Utsunomiya Y.T., Neves H.H., Gomes R.C., Garcia J.F., Fukumasu H., Silva S.L., Junior G.A.O., Alexandre P.A., Leme P.R., et al 2014 Genome-wide association analysis of feed intake and residual feed intake in Nellore cattle. BMC genetics 15(1):21. 24517472 10.1186/1471-2156-15-21 PMC3925773

[CIT0029] Silva R.M., Fragomeni B.O., Lourenco D.A., Magalhães A.F., Irano N., Carvalheiro R., Canesin R.C., Mercadante M.E., Boligon A.A., Baldi F.S., et al. 2016 Accuracies of genomic prediction of feed efficiency traits using different prediction and validation methods in an experimental Nelore cattle population. J. Anim. Sci. 94:3613–3623. doi:10.2527/jas.2016-0401. 27898889

[CIT0030] Torres-Junior R., Silva L.O.C., Favero R., Gomes R.C., Gondo A., Tsuruta S., Costa M.V., Okamura V., Menezes G.R.O., Nobre P.R.C., et al 2018 Is a 35-day feeding test with automatic daily weighting good enough for evaluating beef cattle for feed efficiency traits? In: ICAR Conference, Auckland, New Zealand.

[CIT0031] Yáñez J. M., Houston R. D., and Newman S. 2014 Genetics and genomics of disease resistance in salmonid species. Front. Genet. 5:415. doi:10.3389/fgene.2014.00415. 25505486 PMC4245001

